# Differences in adverse drug reactions between GnRH antagonists and agonists in the treatment of prostate cancer: a retrospective analysis using real-world data

**DOI:** 10.3389/fphar.2026.1895503

**Published:** 2026-08-18

**Authors:** Yingxun Luo, Xuwei Hong, Weiqiang Lin, Hong Huang, Jiawei Wang, Zeyao Xu, Weinan Zeng, Yuanfeng Zhang, Yonghai Zhang

**Affiliations:** 1 Department of Urology, Shantou Central Hospital, Shantou, China; 2 Shantou Key Laboratory of Basic and Translational Research of Malignant Tumor, Shantou, China; 3 Department of Nephrology, Shantou Central Hospital, Shantou, China

**Keywords:** adverse drug reaction, GnRH agonist, GnRH antagonist, prostate cancer, real-world study

## Abstract

**Purpose:**

This study aimed to evaluate the safety profiles of gonadotropin-releasing hormone (GnRH) antagonists and agonists in the treatment of prostate cancer (PC).

**Methods:**

A retrospective pharmacovigilance analysis was conducted using the US Food and Drug Administration Adverse Event Reporting System (FAERS) database from the first quarter of 2004 to the fourth quarter of 2024. Within-class direct comparison analyses between GnRH antagonists and GnRH agonists were performed using disproportionality methods, including the Reporting Odds Ratio (ROR) and Proportional Reporting Ratio (PRR). Further analyses on age stratification, combination therapy, and onset time of adverse events (AEs) were conducted to explore the safety differences between GnRH antagonists and agonists.

**Results:**

A total of 2,486 reports were included in the study, comprising 444 reports for GnRH antagonists and 2,042 reports for GnRH agonists. GnRH antagonists showed higher risk for injection site reactions compared with GnRH agonists, with the strongest signals observed for injection site erythema (ROR = 16.07, 95% confidence interval [CI] = 9.14–28.28; PRR = 14.33; χ^2^ = 159.30), injection site pain (ROR = 10.44, 95% CI = 6.53–16.71; PRR = 9.27; χ^2^ = 140.27), and injection site swelling (ROR = 10.31, 95% CI = 5.58–19.05; PRR = 9.63; χ^2^ = 82.72). Age-stratified analysis demonstrated that injection site reactions remained the predominant signals across age groups, while patients aged ≥65 years exhibited a broader spectrum of AE signals. Time-to-onset analysis revealed different temporal patterns between the two drug classes, with a higher proportion of GnRH antagonist-associated AEs occurring within 30 days after treatment initiation, whereas GnRH agonist-associated AEs were more frequently reported after 360 days. Concomitant medication subgroup analyses further identified distinct AE reporting patterns in specific treatment contexts.

**Conclusion:**

This pharmacovigilance study identified distinct AE reporting patterns between GnRH antagonists and GnRH agonists in PC treatment. GnRH antagonists were primarily characterized by higher risk of local injection site reactions, whereas AE profiles varied according to patient age, treatment duration, and concomitant medication exposure.

## 1 Introduction

Prostate cancer (PC) is the second most common malignant tumor in men worldwide. In 2021, there were approximately over 10 million PC patients globally, and the prevalence is expected to continue increasing over the next 25 years (Zhao et al., 2025). Androgen deprivation therapy (ADT) is the standard treatment for intermediate-risk PC (Desai et al., 2021). Among ADT regimens, gonadotropin-releasing hormone (GnRH) modulators serve as first-line agents, which are categorized into two main classes: GnRH agonists and GnRH antagonists (Shahinian et al., 2010). Both types of drugs regulate the hypothalamic-pituitary-gonadal axis to reduce serum testosterone levels and inhibit tumor growth; however, significant differences exist in the occurrence of adverse drug reactions (ADRs) during clinical application, which affect treatment outcomes in patients.

GnRH agonists activate pituitary GnRH receptors, leading to increased secretion of luteinizing hormone (LH) and follicle-stimulating hormone (FSH). A rebound elevation in testosterone occurs 1–2 weeks after initial administration, potentially inducing adverse reactions such as exacerbated bone pain, urinary tract obstruction, and even spinal cord compression (2013; Van Poppel and Abrahamsson, 2020; Liu et al., 2021; Raja et al., 2022). Although combined use with nonsteroidal antiandrogens can alleviate the initial rebound, long-term ADRs still significantly compromise patient adherence (Tsushima et al., 2001). In contrast, GnRH antagonists competitively block pituitary GnRH receptors, rapidly inhibiting LH and FSH release, avoiding transient testosterone surges, and reducing the risk of acute initial adverse events (AEs) (Uttley et al., 2017). A systematic review has confirmed that GnRH antagonists exhibit superior long-term cardiovascular safety compared to GnRH agonists [relative risk, (RR) = 0.66, 95%CI: 0.53–0.82] (Gu et al., 2023). Nevertheless, a study has indicated a high incidence of injection site reactions with subcutaneous degarelix (Barkin et al., 2016). And a case report documented pituitary apoplexy following treatment with GnRH agonists for PC (Elshimy et al., 2022). However, the comparative safety profiles of GnRH antagonists and GnRH agonists have not been fully characterized, and large-scale real-world analyses evaluating differences in ADR patterns between these two drug classes remain lacking.

The U.S. Food and Drug Administration Adverse Event Reporting System (FAERS) is one of the world’s largest public spontaneous ADR reporting databases, encompassing diverse patient populations and ADR types in real-world practice. It provides an ideal data source for comparing ADR differences between GnRH agonists and antagonists. Therefore, this study conducted a retrospective analysis using the FAERS database to systematically compare the differences and characteristics of ADRs associated with GnRH agonists versus antagonists in the treatment of PC, aiming to provide evidence-based support for clinical selection of appropriate patient populations and optimization of ADT regimens.

## 2 Methods

### 2.1 Study design and data source

This was a retrospective study with data derived from FAERS, a comprehensive database that collects AE reports, medication error reports, and product quality complaints leading to AEs (Zhang et al., 2021). In this study, American Standard Code for Information Interchange (ASCII) data files were obtained from the FAERS database (https://open.fda.gov/apis/drug/) covering the period from the first quarter of 2004 to the fourth quarter of 2024.

### 2.2 Inclusion and exclusion criteria

The inclusion criteria were as follows: (1) Diagnosed as PC (C61) according to the International Classification of Diseases, Tenth Revision (ICD-10); (2) Patients who had received treatment for PC with GnRH antagonists (e.g., degarelix, abarelix, etc.) or GnRH agonists (e.g., leuprorelin, goserelin, triptorelin, etc.); (3) Officially recorded AE reports in the FAERS database, with extracted information such as patient sex, age at AE occurrence, and outcomes; (4) AEs recorded in the reports had a clear temporal correlation with the use of GnRH antagonists or GnRH agonists.

Exclusion criteria were as follows: (1) Duplicate reports of the same AE from the same patient were excluded, and only the first report data were retained; (2) Data missing two or more key items among basic patient information, medication information, and AE information were excluded; (3) Data with ambiguous key information such as drug names and AE descriptions in the reports were excluded.

### 2.3 AE definition

FAERS uses the Medical Dictionary for Regulatory Activities (MedDRA) to standardize adverse reactions caused by GnRH antagonists or GnRH agonists. This study focused primarily on the Preferred Term (PT) level.

### 2.4 Statistical analysis

Disproportionality analysis was performed to detect potential adverse drug reaction (ADR) signals. The Reporting Odds Ratio (ROR) and Proportional Reporting Ratio (PRR) methods were used for signal detection. A within-class direct comparison analysis of FAERS reports was conducted to compare AE reporting patterns between GnRH agonists and GnRH antagonists. The 2 × 2 contingency table and positive signal detection thresholds for the disproportionality analyses are provided in Supplementary Tables 1, 2, respectively.

In the direct comparison analysis, an ROR >1 indicated that the target AE was relatively more frequently reported with GnRH antagonists than with GnRH agonists, whereas an ROR <1 suggested a relatively higher reporting proportion of the target AE with GnRH agonists. For the ROR method, a potential signal was considered detected when the number of reports was ≥3 and the lower limit of the 95% confidence interval (CI) of the ROR was ≥1. For the PRR method, a positive signal was defined as PRR ≥2 with a corresponding χ^2^ value ≥ 4. An AE was considered a positive signal when both ROR and PRR criteria were simultaneously met.

For categorical variables such as gender, AE type, and medication type, frequency and percentage were used for statistical description. For continuous variables such as patient age, were summarized using median and interquartile range (IQR). Time to onset (TTO) was defined as the interval between the date of AE onset (EVENT_DT) and the date of drug administration (START_DT). Subgroup analyses stratified by age were performed to further evaluate potential differences in AE reporting patterns. Data analysis was performed using R software (version 4.4.3), and the significance level α was set at 0.05. *P* values were adjusted for multiple comparisons using the false discovery rate (FDR) method.

## 3 Results

### 3.1 Descriptive analysis

**FIGURE 1:**
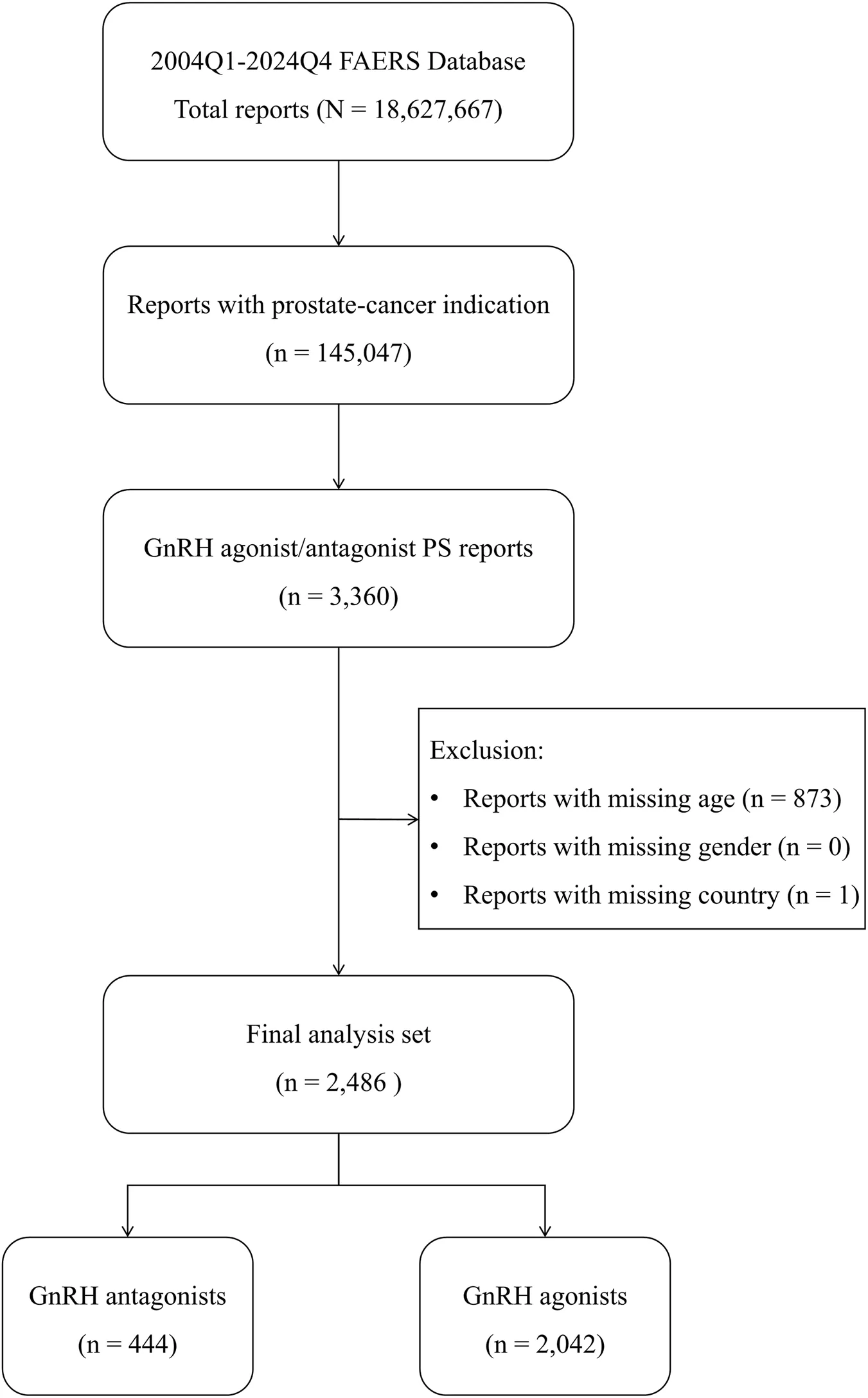
Flowchart of GnRH antagonists and GnRH agonists in FAERS.

From the first quarter of 2004 to the fourth quarter of 2024, a total of 18,627,667 AE reports were collected by FAERS. After applying inclusion and exclusion criteria, 2,486 reports were finally included, of which 2,042 were associated with GnRH agonists and 444 with GnRH antagonists (Figure 1). The majority of patients receiving GnRH agonists or GnRH antagonists were aged 65–85 years. AE reports for GnRH agonists mainly originated from consumers (20.3%) and medical doctors (19.9%), while those for GnRH antagonists were mostly submitted by medical doctors (17.6%) and other personnel (34.2%). The mortality rate was significantly higher in patients treated with GnRH agonists than with GnRH antagonists (36.1% vs. 11.9%, *P* < 0.001); however, the proportion of hospitalizations attributed to GnRH antagonists was higher (47.1% vs. 30.9%). AE reports for both GnRH agonists and antagonists were largely from Japan, accounting for 29.4% and 40.1% respectively (Table 1).

**TABLE 1:** Baseline characteristics of FAERS reports in the revised main analysis set.

Variable	Overall (N = 2,486)	GnRH agonists (N = 2,042)	GnRH antagonists (N = 444)	*P* value
Age, n (%)	​	​	​	0.148
≤17 years	5 (0.2)	4 (0.2)	1 (0.2)	​
18–64 years	276 (11.1)	213 (10.4)	63 (14.2)	​
65–85 years	1,892 (76.1)	1,564 (76.6)	328 (73.9)	​
≥86 years	313 (12.6)	261 (12.8)	52 (11.7)	​
Weight, n (%)	​	​	​	0.115
<50 kg	38 (6.0)	21 (4.9)	17 (8.2)	​
>100 kg	54 (8.5)	41 (9.6)	13 (6.2)	​
50–100 kg	541 (85.5)	363 (85.4)	178 (85.6)	​
Reporter type, n (%)	​	​	​	<0.001
CN	461 (18.5)	414 (20.3)	47 (10.6)	​
HP	331 (13.3)	313 (15.3)	18 (4.1)	​
MD	485 (19.5)	407 (19.9)	78 (17.6)	​
OT	459 (18.5)	307 (15.0)	152 (34.2)	​
PH	115 (4.6)	99 (4.8)	16 (3.6)	​
RN	7 (0.3)	0 (0.0)	7 (1.6)	​
Missing	628 (25.3)	502 (24.6)	126 (28.4)	​
Outcome, n (%)	​	​	​	<0.001
DE	790 (31.8)	737 (36.1)	53 (11.9)	​
LT	86 (3.5)	74 (3.6)	12 (2.7)	​
HO	839 (33.7)	630 (30.9)	209 (47.1)	​
DS	41 (1.6)	39 (1.9)	2 (0.5)	​
CA	0 (0.0)	0 (0.0)	0 (0.0)	​
RI	2 (0.1)	2 (0.1)	0 (0.0)	​
OT	563 (22.6)	466 (22.8)	97 (21.8)	​
Missing	165 (6.6)	94 (4.6)	71 (16.0)	​
Reporter country, n (%)	​	​	​	<0.001
Japan	778 (31.3)	600 (29.4)	178 (40.1)	​
Canada	286 (11.5)	184 (9.0)	102 (23.0)	​
United States	244 (9.8)	146 (7.1)	98 (22.1)	​
Netherlands	210 (8.4)	204 (10.0)	6 (1.4)	​
France	116 (4.7)	109 (5.3)	7 (1.6)	​
Other	500 (20.1)	449 (22.0)	51 (11.5)	​
Not specified	352 (14.2)	350 (17.1)	2 (0.5)	​

AE: adverse event; CN: consumer; HP: health professional; MD: medical doctor; OT: other; PH: pharmacist; DE: death; DS: disability; HO: hospitalization; LT: life-threatening.

### 3.2 PT level disproportionality analysis

At the PT level, disproportionality analysis identified 12 positive signals (Table 2). Injection site-related AEs showed the strongest disproportional reporting signals. Among them, injection site erythema demonstrated the highest signal strength (ROR = 16.07, 95% CI = 9.14–28.28; PRR = 14.33; χ^2^ = 159.30), followed by injection site pain (ROR = 10.44, 95% CI = 6.53–16.71; PRR = 9.27; χ^2^ = 140.27) and injection site swelling (ROR = 10.31, 95% CI = 5.58–19.05; PRR = 9.63; χ^2^ = 82.72). Other injection site-related signals included injection site induration (ROR = 3.76, 95% CI = 2.22–6.38; PRR = 3.60; χ^2^ = 27.50).

**TABLE 2:** Top 30 most frequently reported PTs in the direct within-class comparison between GnRH antagonists and agonists.

PT	Antagonists, n	Agonists, n	Direct ROR (95% CI)	PRR (χ2)	*P* value	FDR *P*
Death	11	256	0.18 (0.1–0.34)	0.21 (37.8)	<0.001	<0.001
Prostate cancer	17	138	0.56 (0.34–0.94)	0.58 (5.04)	0.025	0.242
Prostatic specific antigen increased	16	114	0.65 (0.38–1.1)	0.66 (2.64)	0.105	0.645
Hot flush	14	86	0.76 (0.43–1.34)	0.77 (0.89)	0.345	0.938
Asthenia	28	67	2 (1.28–3.14)	1.94 (9.47)	0.002	0.074
Fatigue	22	72	1.45 (0.89–2.35)	1.42 (2.26)	0.133	0.67
Pneumonia	15	75	0.94 (0.54–1.64)	0.94 (0.05)	0.829	0.957
Injection site pain *	55	27	10.44 (6.53–16.71)	9.27 (140.27)	<0.001	<0.001
Fall	15	67	1.06 (0.6–1.85)	1.05 (0.04)	0.849	0.957
Interstitial lung disease	18	56	1.53 (0.89–2.6)	1.5 (2.42)	0.12	0.67
Metastases to bone	10	62	0.77 (0.4–1.48)	0.77 (0.63)	0.428	0.938
Injection site erythema *	51	16	16.07 (9.14–28.28)	14.33 (159.3)	<0.001	<0.001
Anaemia	15	47	1.52 (0.85–2.72)	1.5 (1.99)	0.159	0.713
Dyspnoea	15	46	1.55 (0.86–2.78)	1.53 (2.19)	0.139	0.67
Pyrexia *	25	35	3.44 (2.04–5.78)	3.3 (24.36)	<0.001	<0.001
Cardiac failure	9	50	0.86 (0.43–1.73)	0.86 (0.18)	0.675	0.938
Injection site induration *	25	32	3.76 (2.22–6.38)	3.6 (27.5)	<0.001	<0.001
Pain *	26	30	4.18 (2.46–7.11)	3.99 (32.5)	<0.001	<0.001
Nausea *	19	36	2.52 (1.44–4.41)	2.45 (11.19)	<0.001	0.036
Drug ineffective	2	49	0.23 (0.06–0.81)	0.23 (6.19)	0.013	0.242
General physical health deterioration	6	45	0.65 (0.28–1.49)	0.66 (1.05)	0.306	0.938
Malaise *	19	30	3.02 (1.7–5.39)	2.94 (15.49)	<0.001	0.006
Injection site swelling *	32	15	10.31 (5.58–19.05)	9.63 (82.72)	<0.001	<0.001
Decreased appetite *	22	25	4.21 (2.37–7.5)	4.05 (28.01)	<0.001	<0.001
Malignant neoplasm progression	7	39	0.87 (0.4–1.91)	0.87 (0.12)	0.728	0.938
Back pain *	14	31	2.15 (1.14–4.04)	2.11 (5.93)	0.015	0.242
Condition aggravated	11	33	1.59 (0.81–3.13)	1.58 (1.84)	0.175	0.777
Vomiting *	19	22	4.12 (2.22–7.61)	3.98 (23.7)	<0.001	<0.001
Cerebral infarction	6	32	0.92 (0.39–2.14)	0.92 (0.04)	0.841	0.957
Diarrhoea *	12	24	2.38 (1.2–4.74)	2.34 (6.46)	0.011	0.242

PTs, were ranked by the total number of event reports. Direct ROR, and PRR, were calculated from the same within-class 2 × 2 table comparing GnRH, antagonists versus GnRH, agonists. * indicate statistically significant signals in the algorithm. ROR, reporting odds ratio; PRR, proportional reporting ratio; PT, preferred term; CI, confidence interval; FDR, false discovery rate.

Several systemic AEs also showed significant disproportional reporting. Positive signals included decreased appetite (ROR = 4.21, 95% CI = 2.37–7.50; PRR = 4.05; χ^2^ = 28.01), pain (ROR = 4.18, 95% CI = 2.46–7.11; PRR = 3.99; χ^2^ = 32.50), vomiting (ROR = 4.12, 95% CI = 2.22–7.61; PRR = 3.98; χ^2^ = 23.70), and pyrexia (ROR = 3.44, 95% CI = 2.04–5.78; PRR = 3.30; χ^2^ = 24.36). Additional positive signals were observed for malaise (ROR = 3.02, 95% CI = 1.70–5.39; PRR = 2.94; χ^2^ = 15.49), nausea (ROR = 2.52, 95% CI = 1.44–4.41; PRR = 2.45; χ^2^ = 11.19), diarrhoea (ROR = 2.38, 95% CI = 1.20–4.74; PRR = 2.34; χ^2^ = 6.46), and back pain (ROR = 2.15, 95% CI = 1.14–4.04; PRR = 2.11; χ^2^ = 5.93).

### 3.3 Subgroup analysis by age

After stratifying the study population by age (≥65 years and <65 years), the signal intensities of PTs for GnRH agonists and antagonists were further analyzed (Table 3).

**TABLE 3:** Age-stratified direct within-class comparison of PT reporting between GnRH antagonists and agonists.

Age subgroup	PT	Antagonists, n	Agonists, n	Direct ROR (95% CI)	PRR (χ2)	FDR *P*
<65 years	Injection site erythema	13	1	37.83 (6.82–210.04)	30.18 (40.23)	<0.001
<65 years	Injection site pain	12	4	11.3 (3.69–34.59)	9.32 (26.13)	<0.001
≥65 years	Injection site erythema	38	15	13.13 (7.2–23.94)	11.9 (113.15)	<0.001
≥65 years	Injection site pain	43	23	9.89 (5.91–16.55)	8.87 (109.9)	<0.001
≥65 years	Injection site swelling	27	14	9.72 (5.09–18.55)	9.09 (69.68)	<0.001
≥65 years	Injection site cellulitis	11	1	37.86 (6.88–208.3)	36.74 (46.41)	<0.001
≥65 years	Pain	24	24	5.05 (2.85–8.95)	4.79 (37.6)	<0.001
≥65 years	Intentional product use issue	8	0	83.31 (4.8–1446.61)	81.48 (37.69)	<0.001
≥65 years	Decreased appetite	21	21	5.02 (2.73–9.22)	4.79 (32.91)	<0.001
≥65 years	Death	11	242	0.2 (0.11–0.37)	0.23 (32.6)	<0.001
≥65 years	Pyrexia	23	29	4 (2.3–6.96)	3.82 (27.87)	<0.001
≥65 years	Injection site warmth	8	2	16.64 (4.04–68.49)	16.3 (27.87)	<0.001
≥65 years	Intentional product misuse	6	0	63.37 (3.56–1127.33)	62.3 (28.1)	<0.001
≥65 years	Abdominal pain	12	8	7.25 (3.01–17.46)	7.05 (26.51)	<0.001
≥65 years	Tachycardia	6	1	21.11 (3.56–125.05)	20.77 (23.02)	<0.001
≥65 years	Blood pressure decreased	9	5	8.46 (2.94–24.34)	8.28 (22.44)	<0.001
≥65 years	Vomiting	16	18	4.42 (2.26–8.67)	4.27 (22.23)	<0.001
≥65 years	Injection site induration	21	30	3.52 (2–6.18)	3.38 (21.62)	<0.001
≥65 years	Dysuria	11	9	5.95 (2.5–14.16)	5.8 (20.87)	<0.001
≥65 years	Chills	7	3	10.46 (2.93–37.37)	10.27 (20.07)	<0.001
≥65 years	Urticaria	8	5	7.55 (2.57–22.2)	7.41 (18.62)	0.001
≥65 years	Erythema	7	5	6.65 (2.2–20.09)	6.54 (14.96)	0.007
≥65 years	Injection site Mass	7	5	6.65 (2.2–20.09)	6.54 (14.96)	0.007
≥65 years	Injection site discomfort	5	2	10.68 (2.39–47.84)	10.54 (14.9)	0.007
≥65 years	Malaise	17	29	2.93 (1.61–5.35)	2.84 (13.41)	0.013
≥65 years	Dehydration	8	8	4.88 (1.87–12.71)	4.79 (12.85)	0.017
≥65 years	Shock	5	3	7.63 (1.99–29.27)	7.53 (12.16)	0.024
≥65 years	Loss of consciousness	10	13	3.8 (1.69–8.59)	3.73 (11.92)	0.026
≥65 years	Feeling abnormal	6	5	5.74 (1.84–17.98)	5.66 (11.5)	0.032

This age-stratified analysis was considered exploratory. PTs, were retained when at least one comparison group had ≤5 reports for the PT, the 95% CI, did not cross 1, and the FDR-adjusted P value was <0.05. PT, preferred term; ROR, reporting odds ratio; PRR, proportional reporting ratio; CI, confidence interval.

In patients aged <65 years, two positive signals were identified. Injection site erythema showed the strongest disproportional reporting signal (ROR = 37.83, 95% CI = 6.82–210.04; PRR = 30.18; χ^2^ = 40.23; FDR-adjusted *P* < 0.001), followed by injection site pain (ROR = 11.30, 95% CI = 3.69–34.59; PRR = 9.32; χ^2^ = 26.13; FDR-adjusted *P* < 0.001).

Among patients aged ≥65 years, injection site-related events remained the predominant signals, with strong associations observed for injection site erythema (ROR = 13.13, 95% CI = 7.20–23.94; PRR = 11.90; χ^2^ = 113.15; FDR-adjusted *P* < 0.001), injection site pain (ROR = 9.89, 95% CI = 5.91–16.55; PRR = 8.87; χ^2^ = 109.90; FDR-adjusted *P* < 0.001), injection site swelling (ROR = 9.72, 95% CI = 5.09–18.55; PRR = 9.09; χ^2^ = 69.68; FDR-adjusted *P* < 0.001), and injection site cellulitis (ROR = 37.86, 95% CI = 6.88–208.30; PRR = 36.74; χ^2^ = 46.41; FDR-adjusted *P* < 0.001). Several systemic AEs, including pain, decreased appetite, pyrexia, and vomiting, also showed positive signals in this subgroup. These AEs were relatively more frequently reported with GnRH antagonists than with GnRH agonists.

### 3.4 Analysis of AEs in combination therapy

The distribution of concomitant medication categories by GnRH drug class is summarized in Supplementary Table 3. Cardiovascular drugs were the most frequently reported concomitant medications in both GnRH antagonists (129/444, 29.05%) and GnRH agonists (381/2042, 18.66%). These concomitant medication subgroups were further evaluated using within-class disproportionality analyses, and significant signals are presented in Table 4. Positive signals identified in the subgroup analyses were consistent with the overall findings, with injection site reactions remaining the predominant disproportional reporting signals.

**TABLE 4:** Concomitant medication direct within-class comparison of PT reporting in concomitant medication subgroups.

Concomitant category	PT	Antagonists, n	Agonists, n	Direct ROR (95% CI)	PRR (χ2)	FDR *P*
Cardiovascular drugs	Injection site erythema	25	5	16.70 (6.48–43.05)	13.62 (56.33)	<0.001
Cardiovascular drugs	Injection site pain	22	6	12.09 (4.92–29.70)	10.17 (44.21)	<0.001
Cardiovascular drugs	Injection site swelling	19	3	19.08 (6.00–60.72)	16.37 (44.84)	<0.001
Abiraterone	Decreased appetite	2	3	20.71 (2.65–161.75)	10.86 (14.10)	0.004
Enzalutamide	Malaise	6	1	39.90 (6.23–255.33)	27.86 (33.26)	<0.001
Enzalutamide	Intentional product misuse	3	0	53.80 (2.67–1085.81)	45.00 (19.32)	0.002
Enzalutamide	Decreased appetite	5	4	10.29 (2.66–39.80)	7.86 (15.83)	0.006
Enzalutamide	Nausea	5	4	10.29 (2.66–39.80)	7.86 (15.83)	0.006

Concomitant medication categories were defined at the report level and were not mutually exclusive. Cardiovascular drugs included prespecified cardiovascular medication terms. PTs, in panel B were retained when the FDR-adjusted P value was <0.05 in the direct within-class comparison within a concomitant medication subgroup. Percentages in panel A use the total number of reports in each GnRH, class as the denominator, whereas percentages in panel B use the number of reports in the corresponding concomitant medication subgroup. PT, preferred term; ROR, reporting odds ratio; PRR, proportional reporting ratio; FDR, false discovery rate; CI, confidence interval.

Among reports involving concomitant cardiovascular drugs, three injection site-related events showed significantly higher reporting frequencies with GnRH antagonists than with GnRH agonists, including injection site swelling (ROR = 19.08, 95% CI = 6.00–60.72; PRR = 16.37; χ^2^ = 44.84; FDR-adjusted *P* < 0.001), injection site erythema (ROR = 16.70, 95% CI = 6.48–43.05; PRR = 13.62; χ^2^ = 56.33; FDR-adjusted *P* < 0.001), and injection site pain (ROR = 12.09, 95% CI = 4.92–29.70; PRR = 10.17; χ^2^ = 44.21; FDR-adjusted *P* < 0.001).

In patients receiving concomitant abiraterone, decreased appetite was identified as a significant signal (ROR = 20.71, 95% CI = 2.65–161.75; PRR = 10.86; χ^2^ = 14.10; FDR-adjusted *P* = 0.004). Among reports with concomitant enzalutamide use, several systemic AEs showed significant disproportional reporting, including malaise (ROR = 39.90, 95% CI = 6.23–255.33; PRR = 27.86; χ^2^ = 33.26; FDR-adjusted *P* < 0.001), intentional product misuse (ROR = 53.80, 95% CI = 2.67–1085.81; PRR = 45.00; χ^2^ = 19.32; FDR-adjusted *P* = 0.002), decreased appetite (ROR = 10.29, 95% CI = 2.66–39.80; PRR = 7.86; χ^2^ = 15.83; FDR-adjusted *P* = 0.006), and nausea (ROR = 10.29, 95% CI = 2.66–39.80; PRR = 7.86; χ^2^ = 15.83; FDR-adjusted *P* = 0.006).

### 3.5 Onset time of AEs

**FIGURE 2:**
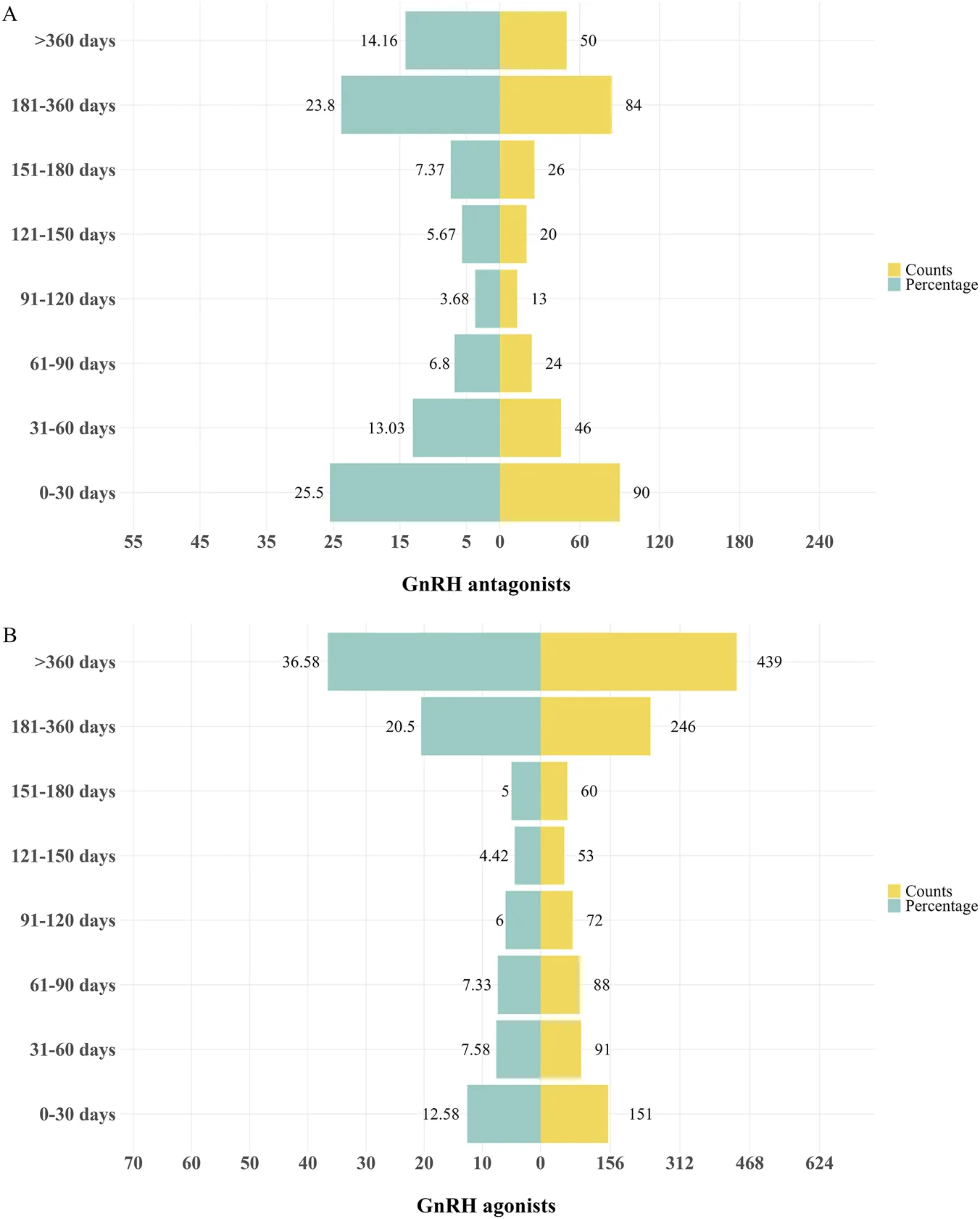
Temporal distribution of PT-level AE onset post-administration of GnRH antagonists **(A)** and GnRH agonists **(B)**.

As shown in Figure 2A, most AEs of GnRH antagonists occurred within 0–30days, accounting for 25.5%. The lowest probability of occurrence was observed during the 91–120-day period (3.68%). Notably, 14.16% of AEs occurred more than 360 days after GnRH antagonist administration. However, Notably, 14.16% of AEs occurred more than 360 days after GnRH antagonist administration (Figure 2B). The lowest proportion of AE onset was observed during the 121–150-day period (4.42%). 12.58% of AEs occurred within 0–30 days after GnRH agonist administration.

## 4 Discussion

This study systematically compared the adverse reaction profiles, population characteristics, and combination therapy safety of GnRH antagonists and agonists in the treatment of PC based on real-world data from the FAERS database. It revealed the distribution patterns and clinical characteristic differences of adverse reactions between the two classes of drugs in a large-scale real-world setting, providing evidence-based support for optimizing individualized medication strategies for ADT.

At the PT level, positive signals associated with GnRH antagonists were predominantly related to injection site reactions. Injection site erythema, injection site pain, and injection site swelling showed the strongest disproportional reporting signals compared with GnRH agonists. Consistent with previous studies, injection site reactions have been identified as one of the main AEs of GnRH antagonists, potentially attributed to differences in administration methods and injection volumes (Abufaraj et al., 2021; Freedland and Abrahamsson, 2021). Although these events are generally manageable, clinicians should provide appropriate patient education and implement standardized injection procedures to improve treatment tolerability.

Age-stratified analysis further demonstrated that injection site reactions remained the predominant signals across age groups, suggesting that these events were consistently associated with GnRH antagonist therapy regardless of age. However, patients aged ≥65 years exhibited a broader spectrum of positive AE signals compared with younger patients, including systemic symptoms such as pain, decreased appetite, pyrexia, and vomiting. These findings highlight the importance of enhanced safety surveillance in older patients receiving ADT, who often have a higher burden of comorbidities and may be more vulnerable to treatment-related adverse outcomes (Gheorghe et al., 2021).

The analysis of TTO revealed different temporal patterns between GnRH antagonists and GnRH agonists. A higher proportion of AEs associated with GnRH antagonists occurred within the first 30 days after treatment initiation, whereas GnRH agonists showed a greater proportion of late-onset AE reports occurring more than 360 days after administration. AE 1 year later may reflect cumulative treatment-related toxicity (Ghiblawi et al., 2026). These findings suggest that clinical monitoring strategies may be tailored according to the therapeutic class. For GnRH antagonists, early follow-up after initiation may facilitate timely recognition and management of injection-related or acute adverse reactions.

Concomitant medication subgroup analyses provided additional insights into the safety profiles of GnRH therapies under different treatment contexts. Among patients receiving concomitant cardiovascular medications, injection site reactions remained the predominant signals associated with GnRH antagonists (Yang et al., 2025). Signals were observed in specific combination therapy subgroups, including decreased appetite in reports involving abiraterone and malaise, nausea, and decreased appetite in reports involving enzalutamide (Koroki et al., 2021; Yokomizo et al., 2022). However, these findings should be interpreted cautiously because subgroup analyses based on spontaneous reports may be affected by differences in patient characteristics, treatment duration, and reporting behavior.

Compared with previous clinical trials and observational studies, this FAERS-based analysis provides a broad real-world perspective on comparative safety patterns between GnRH antagonists and GnRH agonists. The findings reinforce that GnRH antagonists are characterized mainly by local injection-related AEs, whereas the AE reporting spectrum varies according to patient age and concomitant treatment exposure. These results may assist clinicians in balancing treatment benefits and safety considerations when selecting ADT strategies, particularly in patients requiring long-term therapy or complex combination regimens.

This study has several limitations. Firstly, as a retrospective analysis of AE reports in the FAERS database, the findings may be influenced by the inherent limitations of spontaneous reporting systems, including underreporting, stimulated reporting, and incomplete clinical information. In addition, FAERS does not provide reliable estimates of drug exposure or the total number of treated patients; therefore, the incidence rates of AEs could not be calculated, and the identified signals should be interpreted as differences in reporting patterns rather than absolute risks. Secondly, disproportionality analysis only provides evidence of an association between target drugs and AEs, but cannot establish a causal relationship. The TTO analysis should be interpreted cautiously, as FAERS does not provide complete information on treatment duration, follow-up, or clinically verified onset timing. Thirdly, residual confounding may exist, particularly confounding by indication, as treatment selection may be influenced by patient characteristics, disease severity, comorbidities, and prior therapies. Fourthly, spontaneous reports in FAERS are subject to geographic variations in reporting practices, regulatory environments, and healthcare systems, with a substantial proportion of reports originating from countries such as the United States and Japan, which may limit the generalizability of the findings. Future studies should integrate database data with detailed clinical information and conduct prospective research to further verify the association and causal relationship between GnRH antagonists/agonists and AEs.

## 5 Conclusion

Based on real-world data from the FAERS database, this study identified distinct AE reporting patterns between GnRH antagonists and GnRH agonists in the treatment of prostate cancer. Compared with GnRH agonists, GnRH antagonists showed higher reporting odds for injection site reactions, including injection site erythema, pain, and swelling. Age-stratified and time-to-onset analyses further demonstrated differences in AE reporting patterns across patient populations and treatment periods. Concomitant medication subgroup analyses provided additional insights into the influence of different treatment contexts on AE reporting profiles. These findings improve the understanding of the comparative safety profiles of GnRH antagonists and GnRH agonists and may support individualized safety monitoring and treatment decision-making during androgen deprivation therapy.

## Data Availability

The original contributions presented in the study are included in the article/Supplementary Material, further inquiries can be directed to the corresponding authors.

## References

[B1] Abufaraj M. Iwata T. Kimura S. Haddad A. Al-Ani H. Abusubaih L. (2021). Differential impact of gonadotropin-releasing hormone antagonist *versus* agonist on clinical safety and oncologic outcomes on patients with metastatic prostate cancer: a meta-analysis of randomized controlled trials. Eur. Urol. 79 (1), 44–53. 10.1016/j.eururo.2020.06.002 32605859

[B2] Barkin J. Burton S. Lambert C. (2016). Optimizing subcutaneous injection of the gonadotropin-releasing hormone receptor antagonist degarelix. Can. J. Urol. 23 (1), 8179–8183. 26892063

[B3] Desai K. McManus J. M. Sharifi N. (2021). Hormonal therapy for prostate cancer. Endocr. Rev. 42 (3), 354–373. 10.1210/endrev/bnab002 33480983 PMC8152444

[B4] Elshimy G. Raj R. Jacob A. Correa R. (2022). Late onset of pituitary apoplexy following gonadotropin-releasing hormone agonist for prostate cancer treatment. BMJ Case Rep. 15 (3), e248523. 10.1136/bcr-2021-248523 35256375 PMC8905893

[B5] First-line treatment of metastatic prostate cancer (2013). Androgen suppression for symptomatic disease. Prescrire Int. 22 (135), 48–51. 23444510

[B6] Freedland S. J. Abrahamsson P. A. (2021). Androgen deprivation therapy and side effects: are GnRH antagonists safer? Asian J. Androl. 23 (1), 3–10. 10.4103/aja.aja_22_20 32655041 PMC7831824

[B7] Gheorghe G. S. Hodorogea A. S. Ciobanu A. Nanea I. T. Gheorghe A. C. D. (2021). Androgen deprivation therapy, hypogonadism and cardiovascular toxicity in men with advanced prostate cancer. Curr. Oncol. 28 (5), 3331–3346. 10.3390/curroncol28050289 34590590 PMC8482210

[B8] Ghiblawi A. Mohamed G. Patel T. Connell B. (2026). Association of cardiovascular outcomes with GnRH agonist and antagonist therapy in non-metastatic prostate cancer. J. Clin. Oncol. 44 (16_Suppl. l), 5123. 10.1200/JCO.2026.44.16_suppl.5123

[B9] Gu L. Li X. Liu W. (2023). Adverse cardiovascular effect following gonadotropin-releasing hormone antagonist *versus* GnRH agonist for prostate cancer treatment: a systematic review and meta-analysis. Front. Endocrinol. (Lausanne) 14, 1157857. 10.3389/fendo.2023.1157857 37065739 PMC10102515

[B10] Koroki Y. Imanaka K. Yasuda Y. Harada S. Fujino A. (2021). Safety and efficacy of abiraterone acetate plus prednisolone in patients with castration-resistant prostate cancer: a prospective, observational, post-marketing surveillance study. Jpn. J. Clin. Oncol. 51 (9), 1452–1461. 10.1093/jjco/hyab077 34050660 PMC8405844

[B11] Liu Y. F. Fu S. Q. Yan Y. C. Gong B. B. Xie W. J. Yang X. R. (2021). Progress in clinical research on gonadotropin-releasing hormone receptor antagonists for the treatment of prostate cancer. Drug Des. Devel Ther. 15, 639–649. 10.2147/dddt.S291369 33623372 PMC7896730

[B12] Raja T. Sud R. Addla S. Sarkar K. K. Sridhar P. S. Talreja V. (2022). Gonadotropin-releasing hormone agonists in prostate cancer: a comparative review of efficacy and safety. Indian J. Cancer 59 (Suppl. ment), S142–s159. 10.4103/ijc.IJC_65_21 35343198

[B13] Shahinian V. B. Kuo Y. F. Gilbert S. M. (2010). Reimbursement policy and androgen-deprivation therapy for prostate cancer. N. Engl. J. Med. 363 (19), 1822–1832. 10.1056/NEJMsa0910784 21047226

[B14] Tsushima T. Nasu Y. Saika T. Maki Y. Noda M. Suyama B. (2001). Optimal starting time for flutamide to prevent disease flare in prostate cancer patients treated with a gonadotropin-releasing hormone agonist. Urol. Int. 66 (3), 135–139. 10.1159/000056592 11316974

[B15] Uttley L. Whyte S. Gomersall T. Ren S. Wong R. Chambers D. (2017). Degarelix for treating advanced hormone-dependent prostate cancer: an evidence review group perspective of a NICE single technology appraisal. Pharmacoeconomics 35 (7), 717–726. 10.1007/s40273-016-0481-1 27943135

[B16] Van Poppel H. Abrahamsson P. A. (2020). Considerations for the use of gonadotropin-releasing hormone agonists and antagonists in patients with prostate cancer. Int. J. Urol. 27 (10), 830–837. 10.1111/iju.14303 32662187

[B17] Yang Y. Cui Z. Feng X. Zou F. Wu X. (2025). Post-marketing safety profile of ganirelix in women: a 20-year pharmacovigilance analysis of global adverse drug event databases (2004-2024). BMC Pharmacol. Toxicol. 26 (1), 91. 10.1186/s40360-025-00920-4 40264185 PMC12016398

[B18] Yokomizo A. Yonese J. Egawa S. Fukuhara H. Uemura H. Nishimura K. (2022). Real-world use of enzalutamide in men with nonmetastatic castration-resistant prostate cancer in Japan. Int. J. Clin. Oncol. 27 (2), 418–426. 10.1007/s10147-021-02070-z 34779962 PMC8816761

[B19] Zhang K. W. Reimers M. A. Calaway A. C. Fradley M. G. Ponsky L. Garcia J. A. (2021). Cardiovascular events in men with prostate cancer receiving hormone therapy: an analysis of the FDA adverse event reporting system (FAERS). J. Urol. 206 (3), 613–622. 10.1097/ju.0000000000001785 33872049 PMC10243352

[B20] Zhao X. Liu S. Zou Z. Liang C. (2025). Global, regional, and national prevalence of prostate cancer from 1990 to 2021: a trend and health inequality analyses. Front. Public Health 13, 1595159. 10.3389/fpubh.2025.1595159 40567976 PMC12187607

