# Supplementary material for: Differences in adverse drug reactions between GnRH antagonists and agonists in the treatment of prostate cancer: a retrospective analysis using real-world data
Source: Front Pharmacol. 2026 Aug 18;17:1895503. doi: 10.3389/fphar.2026.1895503 (PMC13529942; doi:10.3389/fphar.2026.1895503)
Supplement: Supplementary file 1 [file Supplementaryfile1.docx]

**Supplementary Files**

**Supplementary Table 1.** **Two-by-two contingency table for direct within-class comparison.**

|  | **Reports with the target PT** | **Reports without the target PT** | **Total** |
| --- | --- | --- | --- |
| GnRH antagonists | a | b | a + b |
| GnRH agonists | c | d | c + d |
| Total | a + c | b + d | a + b + c + d |

Note: a, reports with the target preferred term (PT) among GnRH antagonists; b, reports without the target PT among GnRH antagonists; c, reports with the target PT among GnRH agonists; d, reports without the target PT among GnRH agonists.

**Supplementary Table 2. Signal detection algorithms and predefined criteria.**

| **Method** | **Formula** | **Threshold** |
| --- | --- | --- |
| ROR | $ROR=\frac{ad}{bc}$ | 95% CI (lower limit) > 1, N ≥ 3 |
|  | $95\% CI=e^{ln (ROR)\pm1.96\sqrt{(\frac{1}{a}+\frac{1}{b}+\frac{1}{c}+\frac{1}{d})}}$ |  |
| PRR | $PRR=\frac{a (c+d)}{c (a+b)}$ | PRR ≥ 2, χ^2^ ≥ 4, N ≥ 3 |
|  | $x^{2}=\frac{{(ad-bc)}^{2}\times(a+b+c+d)}{(a+b) (c+d) (a+c) (b+d)}$ |  |

Note: ROR, reporting odds ratio; PRR, proportional reporting ratio; CI, confidence interval.

**Supplementary Table 3.** **Distribution of concomitant medication categories by GnRH drug class.**

| **Concomitant medication category** | **GnRH antagonists, n (%)** | **GnRH agonists, n (%)** |
| --- | --- | --- |
| Cardiovascular drugs | 129 (29.05) | 381 (18.66) |
| Abiraterone | 4 (0.90) | 75 (3.67) |
| Enzalutamide | 20 (4.50) | 134 (6.56) |
| Apalutamide | 1 (0.23) | 20 (0.98) |
